# The Impact of COVID-19 Across Nursing Homes That Disproportionally Serve Minority Residents

**DOI:** 10.1093/geroni/igab046.924

**Published:** 2021-12-17

**Authors:** Maricruz Rivera-Hernandez, Amit Kumar, Indrakshi Roy, Amol Karmarkar, Kimberly Erler, James Rudolph, Julie Baldwin

**Affiliations:** 1 Brown University School of Public Health, Providence, Rhode Island, United States; 2 Northern Arizona University, Flagstaff, Arizona, United States; 3 Sheltering Arms Institute, Richmond, Virginia, United States; 4 MGH Institute of Health Professions, Boston, Massachusetts, United States

## Abstract

The Coronavirus-2019 (COVID-19) pandemic has disproportionally affected communities of color and older adults in the United States. Nursing homes (NHs) have reported over 130,000 COVID-19 deaths (or one-fourth of all US deaths) circa March 2021, a high share of the nation’s total death count (CMS COVID-19 NH Data). These inequities partially driven by barriers to care, segregation and structural racism have resulted in the unequal impact of COVID-19 across NHs (Li et al., 2020). In this presentation, I will describe NHs that disproportionally care for minority residents and the effect of NH composition on COVID-19-related mortality and outcomes. In 2020, minority older adults were less likely to have access to high quality facilities. From June – August, NHs with a high proportion of minority residents reported higher COVID-19 mortality rates per 1000 residents. Equal access to high quality of care across the life-course among racial and ethnic groups is needed.

